# Regional disparities in access to spirometry in the Brazilian public health system

**DOI:** 10.36416/1806-3756/e20250227

**Published:** 2026-03-05

**Authors:** Marcos Otávio Brum Antunes, Jordana Henz Hammes, Jesuély Spieckert de Souza, Mariana Scorsatto Boeira, Simoni Assunção Soares, Frederico Orlando Friedrich, Marcia M M Pizzichini, Marcus Herbert Jones

**Affiliations:** 1. Programa de Pós-Graduação em Pediatria e Saúde da Criança, Pontifícia Universidade Católica do Rio Grande do Sul - PUCRS - Porto Alegre (RS) Brasil.; 2. Programa de Pós-Graduação em Ciências Médicas, Universidade Federal de Santa Catarina - UFSC - Florianópolis (SC) Brasil.

**Keywords:** Respiratory function tests, Public health, Epidemiology

## Abstract

**Objective:**

To describe the spatial distribution of spirometry tests recorded in the Brazilian Sistema Único de Saúde (SUS, Unified Health Care System) in 2023 and 2024.

**Methods:**

This was a population-based observational study analyzing all spirometry records in the SUS Outpatient Database for the 2023-2024 period across all 5,570 Brazilian municipalities. Rates of spirometry per 100,000 population were calculated by using population projections from the Brazilian Institute of Geography and Statistics, considering only individuals ≥ five years of age, and subtracting those with private health insurance-as per data from the National Regulatory Agency for Private Health Insurance and Plans.

**Results:**

A total of 718,464 spirometry tests were recorded in the SUS during the study period (341,654 in 2023 and 376,810 in 2024). The tests were performed in only 398 municipalities (7.1% of the total number of Brazilian municipalities). It is estimated that approximately 83.5 million individuals, representing 61% of the SUS-dependent population, live in municipalities without any records of spirometry. The median distance to the nearest municipality with spirometry was 50 km, being as high as 902 km in remote areas of the Amazon region. The southeastern region had the highest proportion of municipalities providing the test (11.6%), and the northern region had the lowest (2.7%). Among the state capitals, there was marked heterogeneity in test rates, with Vitória at the top (with 2,406 tests per 100,000 population) and Macapá recording no tests.

**Conclusions:**

Access to spirometry in the SUS is highly unequal, with severe geographic and population-based disparities. A significant proportion of the population lacks local availability, often requiring long travel distances to have access to spirometry.

## INTRODUCTION

Spirometry is considered the gold standard for diagnosing and managing various respiratory diseases.^1^ Despite its clinical importance, access to spirometry in Brazil faces complex challenges. The availability of spirometry is limited because Brazil is a vast country with a heterogeneous primary health care system; this is concerning because of the impact of respiratory diseases on the public health system.^2^

Spirometry is essential for diagnosing and monitoring conditions such as asthma and COPD, which place a substantial burden on the public health system.^3^ ^,^ ^4^ In Brazil, the prevalence of physician-diagnosed asthma is estimated at 10% and that of COPD is estimated at 17% among individuals over 40 years of age.^5^ ^,^ ^6^ Underuse of spirometry can compromise early diagnosis, reduce treatment effectiveness, and negatively impact prognosis.^7^

Understanding the spatial dynamics of spirometry tests performed in Brazil will enable the development of more effective strategies for diagnosing and managing pulmonary diseases. Therefore, the objective of the present study was to describe the spatial distribution of spirometry records reported in the country’s public health system.

## METHODS

This was a population-based observational study analyzing spirometry records for the 2023-2024 period. Data were obtained from all 5,570 municipalities in Brazil, thus ensuring comprehensive national coverage for the study period. 

### Data source

The data on spirometry were extracted from the Brazilian *Sistema de Informações Ambulatoriais do Sistema Único de Saúde* (SIA-SUS, Outpatient Information System, Unified Health Care System), available at http://tabnet.datasus.gov.br/cgi/deftohtm.exe?family/cnv/qabr.def), which records outpatient procedures performed in the SUS nationwide. We analyzed pre- and post-bronchodilator spirometry records identified by the code 0211080055, in accordance with the SUS System for the Management System of the Unified Health System Procedure Table, Medications, Orthotics, Prosthetics, and Special Materials (SIGTAP-SUS). The data were collected according to the municipality in which the test was performed^8^

The data on the study population were extracted from the Brazilian Institute of Geography and Statistics Information Retrieval System (available at https://sidra.ibge.gov.br/pesquisa/censo-demografico/series-temporais/series-temporais/) and the National Regulatory Agency for Private Health Insurance and Plans database (available at https://www.ans.gov.br/anstabnet/). The Brazilian Institute of Geography and Statistics Information Retrieval System provides data on the Brazilian population based on the Demographic Census, whereas the National Regulatory Agency for Private Health Insurance and Plans database provides information on supplementary health care, including the number of beneficiaries of private health insurance. 

### Population adjustments

Municipalities in which at least one spirometry test was recorded in either 2023 or 2024 were classified as spirometry providers. 

In order to calculate spirometry rates per 100,000 population, we used interpolated data from the 2010 and 2022 censuses.^9^ Linear regression was used in order to interpolate and estimate the population size for 2023 and 2024 on the basis of the estimated population growth. Only those ≥ 5 years of age were considered, given that spirometry is not indicated for individuals < 5 years of age.^10^ Additionally, beneficiaries of private health insurance were excluded from the total population to ensure that our analysis included only public health system users.^11^

### Data analysis

Descriptive analyses were conducted to characterize the spatial distribution and performance of spirometry tests across all 5,570 Brazilian municipalities in 2023 and 2024. Absolute frequencies and relative proportions of municipalities reporting spirometry were initially calculated and stratified by geographic region to identify potential inequalities in service provision. 

Population-adjusted spirometry rates were estimated by using projected demographic data for 2023 and 2024. Individuals < 5 years of age and those covered by private health insurance were excluded in order to better reflect the population exclusively reliant on the SUS. To ensure complete coverage, population figures from municipalities without spirometry records were included, allowing the analysis to capture service availability and potential gaps in access. Spirometry rates were expressed as the number of tests per 100,000 population, thus allowing regional and temporal comparisons. 

To estimate the population potentially underserved by spirometry, we calculated the number of individuals residing in municipalities without any spirometry records during the study period. This approach assumed that the absence of records reflected a lack of local service provision, although this assumption has limitations. Some individuals may gain access to services in neighboring areas, and inconsistencies in data recording could affect the accuracy of the estimate. Nevertheless, even when services are available in nearby municipalities, individuals without local access would still need to travel to be tested, which may represent a significant barrier to care depending on distance, transportation availability, and socioeconomic conditions. 

Geographic accessibility was assessed by estimating the shortest straight-line distance (geodesic distance) between each municipality without spirometry services and the nearest municipality with recorded spirometry tests. Those distances were calculated between the geographic centroids of municipalities by using methods that consider the Earth’s curvature (i.e., ellipsoidal geometry), being more precise than simple planar calculations. This approach identifies the nearest point on the basis of real geospatial coordinates and returns the minimum spatial separation between each pair of municipalities. The resulting distribution of distances was summarized by using measures of central tendency and dispersion, including median, interquartile range, and extremes. The ten municipalities with the greatest distances to the nearest spirometry provider were also identified.^12^

All data processing and analysis were performed with RStudio, version 4.5.2 (R Foundation for Statistical Computing, Vienna, Austria), the packages *tidyverse*, *sf*, *geobr*, and *nngeo* being used. Data visualizations and spatial linkages were generated from the resulting geospatial structures. Full analytical documentation is available at **https://github.com/mobrant94/spirometry**. 

### Ethics

Because the present study was based on publicly available government data that do not allow identification of the study participants, no research ethics committee approval was required. 

## RESULTS

**Figure 1:**
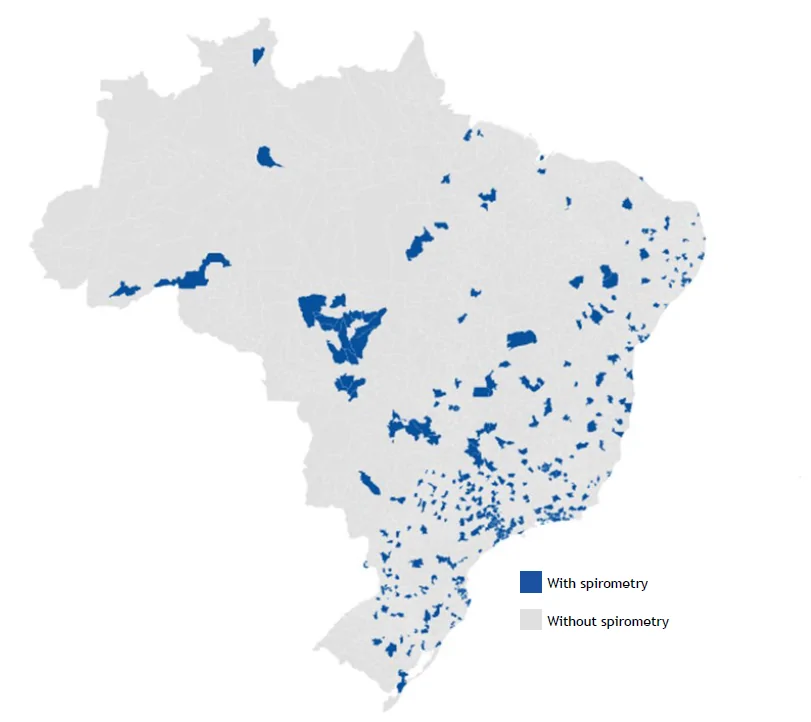
Spatial distribution of recorded spirometry tests in the Brazilian Unified Health Care System in the 2023-2024 period.

In 2023 and 2024, 341,654 and 376,810 spirometry tests, respectively, were recorded in the SUS, totaling 718,464 tests during the study period. Of a total of 5,570 Brazilian municipalities, only 398 (7.1%) reported performing such tests. The national average testing rate was 47 per 100,000 population. Figure 1 displays the spatial distribution of recorded spirometry tests by municipality. 

**Figure 2:**
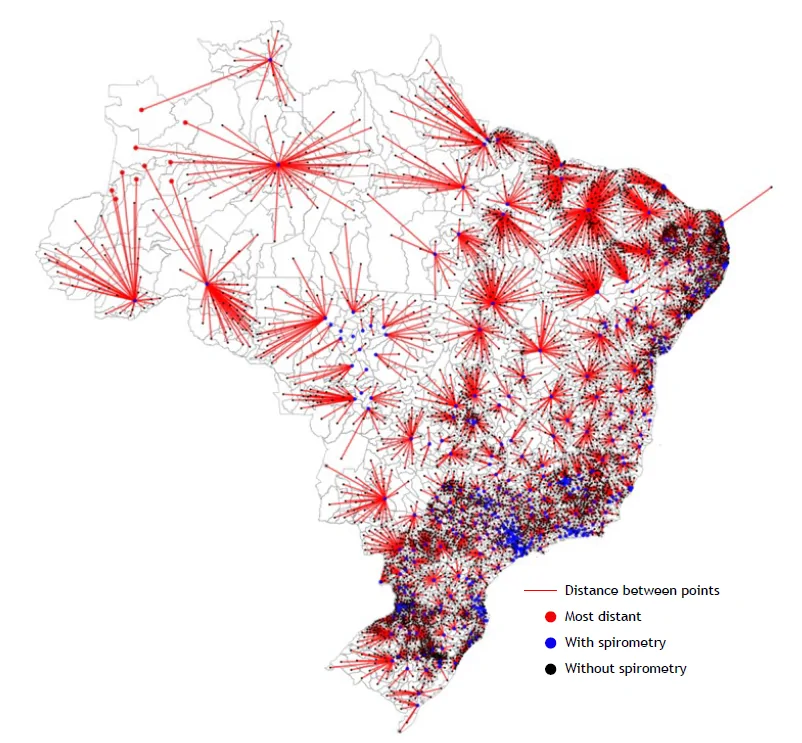
Spatial accessibility to spirometry services in Brazil: municipalities in which spirometry is not available and their nearest referral centers, 2023-2024. Note: Each black dot represents a municipality without any records of spirometry performed in the Brazilian Unified Health Care System in 2023. Blue dots indicate municipalities where spirometry was performed. Red lines connect each municipality without spirometry to its geographically nearest municipality with spirometry, representing potential referral pathways. The largest red dots highlight the two municipalities with the greatest distances to their respective nearest spirometry provider.

Table 1 shows that the highest proportion of municipalities that reported performing the test was in the southeastern region (11.5%), whereas the lowest was in the northern region (2.7%). Despite increases in the number of municipalities that reported performing spirometry in regions such as the central-western region (+23.3%) and the northeastern region (+11.6%), it is estimated that approximately 83.5 million individuals living in areas covered by the SUS still lack access to spirometry, representing approximately 61% of the total number of public health care system users (Figure 2). 

**Table 1:** Regional distribution of municipalities reporting spirometry in the Brazilian Unified Health Care System, as well as estimates of the population without access to spirometry (2023-2024).

Region	Year		Yearly variation (%)	Proportion of municipalities*	Population without access	% of population without access**
	2023	2024				
Central-west	23	30	23.3	7.5	6,615,239	55.7
Northeast	61	69	11.6	4.3	31,073,781	69.9
North	11	11	0.0	2.7	10,291,123	72.3
Southeast	162	180	10.0	11.5	22,563,072	43.7
South	77	75	−2.7	7.0	12,983,825	60.8

*Proportion of municipalities with records of spirometry tests. **Proportion calculated as the Brazilian Unified Health Care System-dependent population ≥ 5 years of age residing in municipalities without spirometry records divided by the total Brazilian Unified Health Care System-dependent population ≥ 5 years of age in each region (with individuals covered by private health insurance being excluded, as adjusted by the National Regulatory Agency for Private Health Insurance and Plans data).

The median distance between municipalities without spirometry services and their nearest provider was 50 km (IQR, 30-90 km). The minimum distance was 3 km, and the maximum distance was 902 km. The ten most geographically distant municipalities from the nearest spirometry provider were all located in the state of Amazonas: Japurá (902 km); São Gabriel da Cachoeira (864 km); Tonantins (814 km); Santo Antônio do Içá (779 km); Amaturá (735 km); Tabatinga (683 km); Fonte Boa (678 km); Juruá (660 km); Santa Isabel do Rio Negro (638 km); and São Paulo de Olivença (626 km). 

**Figure 3:**
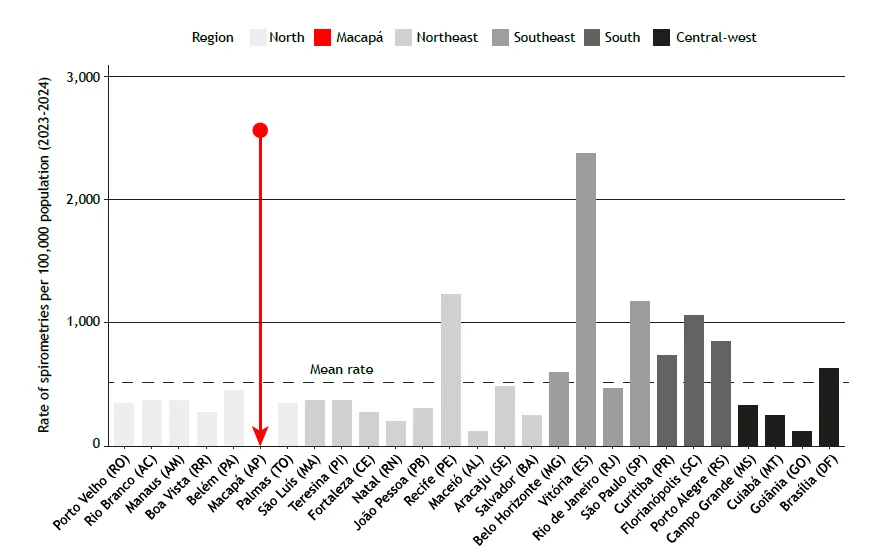
Rate of spirometry tests per 100,000 population in all Brazilian state capitals and the Federal District of Brasília, by region.

Figure 3 highlights the discrepancies in the performance of spirometry across Brazilian capitals. The city of Macapá recorded no tests during the study period. In contrast, the city of Vitória had the highest rate of tests (2,406 per 100,000 population). Other capitals with high rates included Recife, with 1,323 tests per 100,000 population, and São Paulo, with 1,157 tests per 100,000 population. On the other hand, cities such as Goiânia and Maceió had the lowest rates, with 74 tests per 100,000 population and 98 tests per 100,000 population, respectively. 

## DISCUSSION

Our study is the first to investigate the number of municipalities that reported performing spirometry tests in the public health system in Brazil. We found that only 7.1% of the Brazilian municipalities recorded spirometry tests performed in the 2023-2024 period. The southeastern region had the highest proportion of tests (11.5%), whereas the northern region had the lowest (2.7%). The population-adjusted rate for the country as a whole was 47 per 100,000 population. Additionally, 61% of the Brazilian population currently lives in municipalities without access to spirometry in the public health care system. 

Low availability and underutilization of spirometry represent a significant challenge for proper diagnosis and management of respiratory diseases such as asthma and COPD, especially in low- and middle-income countries, where access to spirometry and test interpretation is limited.^13^ ^-^ ^16^ Studies conducted in two Brazilian municipalities showed a low utilization of spirometry, with rates of 5.6% and 9.1% among diagnosed cases of COPD.^17^ These data corroborate the results of the present study, highlighting the underutilization of spirometry in identifying and monitoring respiratory diseases. Moreover, studies have shown high rates of misdiagnosis for diseases such as asthma and COPD, with rates of underdiagnosis ranging from 25.7% to 81.4% and those of overdiagnosis ranging from 28% to 40%.^17^ ^,^ ^18^ Disease identification and control can impact treatment, highlighting the importance of accurate diagnosis and monitoring, which include the performance of spirometry.^19^ ^-^ ^21^

Regional disparities in the performance of spirometry, particularly among the southern, northern, and southeastern regions, may be associated with factors such as access to health care services, availability of trained professionals, and the level of economic development of municipalities. Studies have shown a positive relationship between spirometry performance and consultation with a pulmonologist.^22^ ^,^ ^23^ Data from a study on medical demographics in Brazil show that the southern region has a higher proportion of pulmonologists than does the northern region. However, the southern and southeastern regions have similar specialist-to-population ratios.^24^

The vast territorial extent of the country and the inequalities in access to public health care services represent significant challenges to expanding the availability of spirometry. This scenario may contribute to an increased respiratory disease burden on the public health care system because of inaccurate diagnosis and improper management of cases.^2^ An alarming finding emerged from the present study: no spirometry procedures were recorded in the public health care system of Macapá, the capital of the state of Amapá, or in those of any other municipalities in the state in 2023 or 2024. However, preliminary data from 2025 show the emergence of records, suggesting a potential improvement in service availability.^25^

Regarding regional disparities, an ecological study published in 2024 showed higher rates of hospitalization and mortality from COPD in the southern, central-western, and southeastern regions,^26^ consisting with our findings. This apparent pattern can be explained by greater availability of spirometry in those regions, which likely contributes to better detection and reporting of COPD cases. Consequently, the higher rates of hospitalization and mortality do not necessarily indicate poorer health care performance but may instead reflect more complete identification of severe cases and greater accuracy in outcome recording.^27^ ^,^ ^28^

Factors such as climate differences and socioeconomic inequalities should be considered when evaluating hospitalization rates, mortality, and the performance of spirometry.^29^ The follow-up of patients with respiratory diseases should include spirometry, an essential test to guide the treatment plan, which is based on spirometry results and test interpretation.^19^ COPD is more prevalent in the elderly, and proper performance of spirometry can be challenging in this population because of cognitive and physical limitations. This scenario reinforces the importance of strict control of the technique and proper training of health care professionals.^30^ ^,^ ^31^

Unavailability of spirometry and lack of training for performing the test in many Brazilian cities may compromise early diagnosis of respiratory diseases and hinder proper treatment. Studies indicate that in low- and middle-income countries, limitations in access to spirometry and test interpretation represent significant challenges for the diagnosis and treatment of chronic respiratory diseases.^15^ ^,^ ^16^

The present study showed that there is a marked disparity in the availability of spirometry across Brazilian municipalities, disproportionately favoring more populous areas. This spatial pattern reveals a structural challenge in ensuring equitable access to diagnostic technologies in the SUS, especially in smaller municipalities that often face resource constraints, limited infrastructure, and difficulties in retaining trained professionals. International studies have reported similar scenarios in low- and middle-income countries, where geographic location and population size are strong determinants of access to diagnostic services.^32^ These inequalities may lead to delays in diagnosis, undertreatment, and progression of chronic respiratory conditions such as COPD and asthma. Addressing these gaps requires not only investment in equipment and training but also strengthening of regional care networks capable of integrating and supporting diagnostic capacity in underserved areas.^2^

Access to spirometry is directly linked to access to appropriate treatment, particularly for COPD. The Brazilian National Ministry of Health Clinical Protocols and Therapeutic Guidelines for COPD strongly recommend spirometry because it is essential for confirming the diagnosis and ensuring the necessary documentation for the provision of medications in the SUS.^33^ In this context, spirometry should not be viewed solely as a diagnostic tool but as a key component for enabling timely and evidence-based treatment under optimal conditions.^2^

It is important to highlight that the data analyzed in the present study were obtained from the *Departamento de Informática do SUS* (DATASUS, Information Technology Department of the SUS) and exclusively reflect the procedures recorded in the SUS. Therefore, the numbers do not include tests performed in the private health care sector or those performed as part of study projects or specific initiatives that operate outside the SUS recording systems. This means that the actual volume of spirometry tests performed in Brazil may be higher than that which was reported here.^34^ ^,^ ^35^

In Brazil, programs such as the Telespirometry System Brazil allow performance and interpretation of spirometry in primary health care units located in municipalities with low to medium human development indices through a telehealth platform. Similarly, *RespiraNet*, financially supported by the Rio Grande do Sul State Department of Health, provides remote spirometry-based diagnosis to support the management of chronic respiratory diseases in primary care. However, it is unclear whether the spirometry tests performed through these initiatives are fully captured in the data recorded by the DATASUS.^2^ ^,^ ^36^ Nevertheless, the records extracted from the DATASUS for the present study indicate that access to spirometry is limited for the majority of patients who rely on the public health care system. 

One of the major limitations of the present study is the use of secondary data, with data coverage and completeness being particular issues. However, the SIA-SUS provides a large population-based dataset, with completeness ranging from 90% to 97%, which may mitigate potential data entry errors.^32^ ^,^ ^37^ Additionally, a documented limitation of open databases such as that of the DATASUS is the inconsistency of data collected by third parties. Nevertheless, our experience indicates that the data are reliable and adequately represent the public health care system.^38^ ^,^ ^39^ Approximately 25% of the Brazilian population is covered by private health insurance, meaning that most of the residents in the country rely on the SUS. To better reflect this population, individuals with private coverage were excluded from our population estimates; however, some may still undergo spirometry via the SUS, which could slightly underestimate rates. Moreover, although the quality and completeness of SIA-SUS records may vary across municipalities, the SIA-SUS is a database that is widely used in epidemiological studies because it provides the number of procedures performed and associated costs to the public health system.^11^ ^,^ ^37^ ^,^ ^40^

In recent years, health care initiatives such as expansion of primary care infrastructure, investments in telemedicine, and programs aimed at strengthening respiratory health services have shown the potential to increase access to spirometry and reduce regional disparities.^2^ However, because we used only two years’ worth of cross-sectional data, we were unable to assess temporal trends. Future longitudinal studies using longer historical series could provide valuable insights into whether those policies and investments resulted in improved coverage and availability of spirometry in Brazil. 

In summary, the present study exposed severe limitations in the availability and distribution of spirometry in the Brazilian public health care system. Although the total number of tests increased between 2023 and 2024, only a small fraction of municipalities reported performing spirometry tests, leaving more than half of the SUS-dependent population without access to it. The concentration of services in certain regions, combined with vast distances to the nearest referral centers, especially in the northern region of the country, reflects deep structural and territorial inequities. These barriers probably contribute to the high rates of misdiagnosis and underdiagnosis of chronic respiratory diseases in the country. Expanding access to spirometry and investing in local capacity are essential measures to improve diagnosis, guide treatment, and reduce the burden of respiratory conditions on the public health care system. 
